# Evaluating the Effects of Rivastigmine on Decision-Making in Patients with Mild Cognitive Impairment by Cambridge Neuropsychological Test Automated Battery (CANTAB); A Randomized, Double-Blind, Placebo-Controlled Trial

**DOI:** 10.5812/ijpr-138943

**Published:** 2023-10-30

**Authors:** Setayesh Sadeghi, Fatemeh Mohammadian, Mehdi Tehrani-Doost, Kheirollah Gholami, Niayesh Mohebbi

**Affiliations:** 1 Department of Clinical Pharmacy, Faculty of Pharmacy, Tehran University of Medical Sciences, Tehran, Iran; 2 Department of Psychiatry, Roozbeh Hospital, Tehran University of Medical Sciences, Tehran, Iran; 3 Research Center for Cognitive and Behavioral Sciences, Tehran University of Medial Sciences, Tehran, Iran; 4 Research Center for Rational Use of Drugs, Tehran University of Medical Sciences, Tehran, Iran

**Keywords:** MCI, CANTAB, Decision-making, Rivastigmine, Dementia

## Abstract

**Background:**

Decision-making is a complex process, and most studies showed that patients with mild cognitive impairment (MCI) make worse decisions than healthy people.

**Objectives:**

This study aims to evaluate the effect of rivastigmine on the decision-making of MCI patients using the Cambridge Neuropsychological Test Automated Battery (CANTAB) tests.

**Methods:**

The study was conducted at the Roozbeh Hospital neurology clinic, and 30 patients with mild cognitive impairment over 40 years old were randomly recruited to receive rivastigmine or placebo twice daily for 12 weeks. The initial dose of rivastigmine or placebo was 1.5 mg twice daily and was increased to 3 mg twice daily per patient compliance. A CANTAB test was conducted before and following the intervention.

**Results:**

The mean age of patients in the rivastigmine group was 58.93 ± 10.88, and in the placebo group was 59.33 ± 10.34. The median MMSE (Mini-Mental State Examination) was 26 (IQR = 25 - 26) in both groups. Patients in the rivastigmine group showed significant differences in all subgroup tests of CGT, IST, and SST except in risk adjustment in the CGT test, discrimination in the IST test, and median correct RT on the go trial and SSRT in the SST test. The most commonly reported adverse effects were gastrointestinal complications.

**Conclusions:**

According to the results, rivastigmine significantly improved the primary decision-making outcomes in comparison with placebo.

## 1. Background

Mild cognitive impairment (MCI) is a transient stage between health and Alzheimer's disease (AD) and is categorized into two subgroups, namely amnestic and non-amnestic. In amnestic MCI, impairment in memory is more profound than in other domains, but in non-amnestic MCI, attention and executive function, including planning and decision-making (DM), are more severely affected (1). MCI diagnosis is based on clinical assessment and other tests and evaluations, such as brain imaging. Patients with MCI have subjective and objective complaints in their daily lives. Cognitive impairment can be reversible if underlying modifying risk factors such as depression, hypothyroidism, vitamin B12 deficiency, uncontrolled seizures, cardiovascular risk factors, and traumatic brain injury are present (2-4). On the other hand, MCI develops with increasing age and may lead to dementia in the future. In some studies, the progression rate to AD is estimated at about 10 to 30 percent (5, 6). Decision-making is a complex process, and different factors, such as culture, education, and beliefs, make it more complicated to explain. However, most studies show that people with MCI make worse decisions compared to healthy people in the same situation (7). Financial and health issues are the two most important domains in which patients with MCI make more unacceptable choices because the brain's frontal lobe, which expresses pathological changes in MCI, is involved in such situations (8). In other words, in this population, evidence-based DM shows deviations compared to healthy subjects. Patients with MCI cannot draw an optimal conclusion, so they may show more intensity toward gambling or withdraw from making decisions. Most of the evidence-based DM is related to numerical data, and now it is clear why finance and health are affected in these patients (9, 10).

In the past few decades, various computerized cognitive batteries have been utilized to evaluate cognitive function. These methods have some limitations and opportunities for patients and clinicians; with comprehensive evaluation, clinicians and neuropsychologists can use these batteries with more objective and standardized results. On the other hand, some patients with lower literacy levels may have more difficulties based on these methods (11). The Cambridge Neuropsychological Test Automated Battery (CANTAB) is a test introduced and used in a spacious domain for clinical investigations. CANTAB normalized data was extracted from 2000 subjects aged 4 to 90 who completed the tests. It has 25 tests in 5 domains, including visual memory, executive function, attention, verbal memory, DM and response control, and social cognition. The area of DM in CANTAB has four related tests: the Cambridge Gambling Task (CGT), the Information Sampling Task (IST), the Stop Signal Test (SST), and the Iowa Gambling Task (IGT) (12).

## 2. Objectives

Most previous studies in this population were observational and focused on cognitive and DM differences between healthy people and patients with MCI or AD without any medical intervention. It has been demonstrated that MCI and AD patients with cognitive impairment have worse functions, attention, and DM. Most pharmacologic interventions in clinical studies included donepezil, where they evaluated its effect on gambling (6, 13, 14). It seems there is a lack of controlled trials evaluating pharmacological interventions for DM in patients with MCI by utilizing the CANTAB battery, especially with acetylcholine esterase inhibitors (AChEIs) (15). It would be valuable to find a way to support such patients by preserving or improving their executive functional abilities, DM, and risk-taking behaviors. This encouraged us to design and conduct this study aiming at evaluating the possible effects of rivastigmine on improving the DM of MCI patients with the aid of CANTAB tests.

## 3. Methods

The current study was a prospective, randomized, single-center, double-blind, placebo-controlled clinical trial with parallel groups of MCI patients referred to the Memory Clinic of Roozbeh Hospital in Tehran, Iran, from May 2021 to February 2022. The trial was performed following the principles of the Declaration of Helsinki and the Guidelines for Good Clinical Practice. The institutional review board and ethics committee approved the study protocol, and it was registered on the Clinical Trial Database on May 10, 2021 (IRCT20201104049257N1).

Inclusion Criteria: Patients older than 40 years were eligible for enrollment if they were diagnosed with MCI based on the Diagnostic and Statistical Manual of Mental Disorders, Fifth Edition (DSM-V) criteria, clinical history, and neuropsychological assessment, had a Functional Assessment Staging Tool (FAST) score of three, and consented to participate. The inclusion and exclusion criteria are described in detail in Table 1. Written informed consent was signed by all participants upon enrollment. A neurologist made the diagnosis and performed neurological evaluations.

**Table 1.** Inclusion and Exclusion Criteria of the Study

Inclusion Criteria	Exclusion Criteria
**Patients older than 40 years diagnosed with MCI**	History of major psychiatric disorders
**Consented to participate**	Untreated major depressive disorder
**FAST score: 3**	Vitamin B12 deficiency
**MMSE score: 24 to 30**	Uncontrolled hypothyroidism
**Education: At least third-grade of primary school**	History of drug or alcohol abuse during the past six months
	History of seizures
	Having a major neurologic disorder
	History of stroke
	Consumption of cholinesterase inhibitor agents during the past six months
	Having other diseases that could interfere with decision-making
	Any history of second- or third-degree heart block
	Bradycardia (heart rate< 60 bpm) at the time of enrolment
	GI bleeding during the past six months
	Pregnancy
	Lactation
	History of allergy to rivastigmine
	Participating in another trial

Abbreviations: FAST, functional assessment staging tool; GI, gastrointestinal; MCI, mild cognitive impairmenT; MMSE, mini-mental state examination.

All 30 patients were randomly assigned in blocks of four with a 1:1 allocation ratio to receive either rivastigmine or a matching placebo. All capsules were sealed in an opaque white box. The randomization code and administration were written on the box. The drugs and the placebo were manufactured by Hakim Company. Vitamin B12 deficiency and hypothyroidism were measured by a blood test a month before the study was started. Hypothyroidism was measured by a blood test one month before the start of the study. Other inclusion and exclusion criteria information was extracted from patient medical files and interviews. Brain magnetic resonance imaging (MRI) was performed if the patients did not have brain imaging in their medical records.

In the intervention group, 15 patients were treated with rivastigmine, initially given at 1.5 mg twice daily, then titrated to 3 mg twice a day after two weeks according to tolerability and adherence for 12 weeks. Fifteen patients in the control group received a matching placebo twice daily. A clinical pharmacist blinded to the other parts of the study gave these compounds to the patient.

The patient compliance and adherence to the study protocol were measured by counting pills and an interview. During the first month of the study, the patients were followed every two weeks in terms of increasing the drug dose and watching for possible side effects. After the first month, follow-up visits were held in weeks eight and 12. CANTAB tests that were related to the DM, including CGT, IST, and SST, were conducted by patients at the start of the study and after 12 weeks.

The outcomes were recorded by a computer, and after the completion of the study, all the results were extracted. CGT was used to evaluate the quality and timing of DM as well as the risk tolerance of patients. The CGT output results were shown as the quality of DM, delay aversion, risk-taking, risk adjustment, deliberation time, and overall proportion. According to the CANTAB interpretation results, higher scores in the quality of DM are better. Still, lower scores show better function and self-control for delay aversion, deliberation time, risk-taking, and proportional betting. IST also examines the number of times decisions are modified and the selection speed. SST evaluated reaction time, and the output results of SST showed direction errors on stop and go trials, the proportion of successful stops, the median correct reaction time (RT) on GO trials, stop signal reaction time (SSRT [last half]), and stop signal delay (SSD) at 50% (last half). Unlike the direction errors on stop-and-go trials, higher scores in the proportion of successful stops showed better results, and SSD would be interpreted with other indicators.

For normally distributed variables, the mean and standard deviation (SD) were used to describe the variables. For skewedly distributed variables, the median and the 25th and 75th percentiles were used to describe the variables. A histogram chart and descriptive measures were used to check the normality of the variables. The variables were assessed for differences between the two intervention groups using statistical tests appropriate for the data distribution. Specifically, the independent sample t-test was used for normally distributed variables, while the Mann-Whitney U-test was used for variables with a skewed distribution.

Categorical variables were expressed as frequencies with percentages and compared between the groups using the chi-square test and Fisher’s exact test, as appropriate. The CANTAB test results were analyzed by one variant of the ANCOVA method and were significantly confirmed by P-values less than 0.05. All statistical analyses were conducted using IBM SPSS Statistics for Windows, version 25.0 (Armonk, NY: IBM Corp.).

## 4. Results

**Figure 1.**
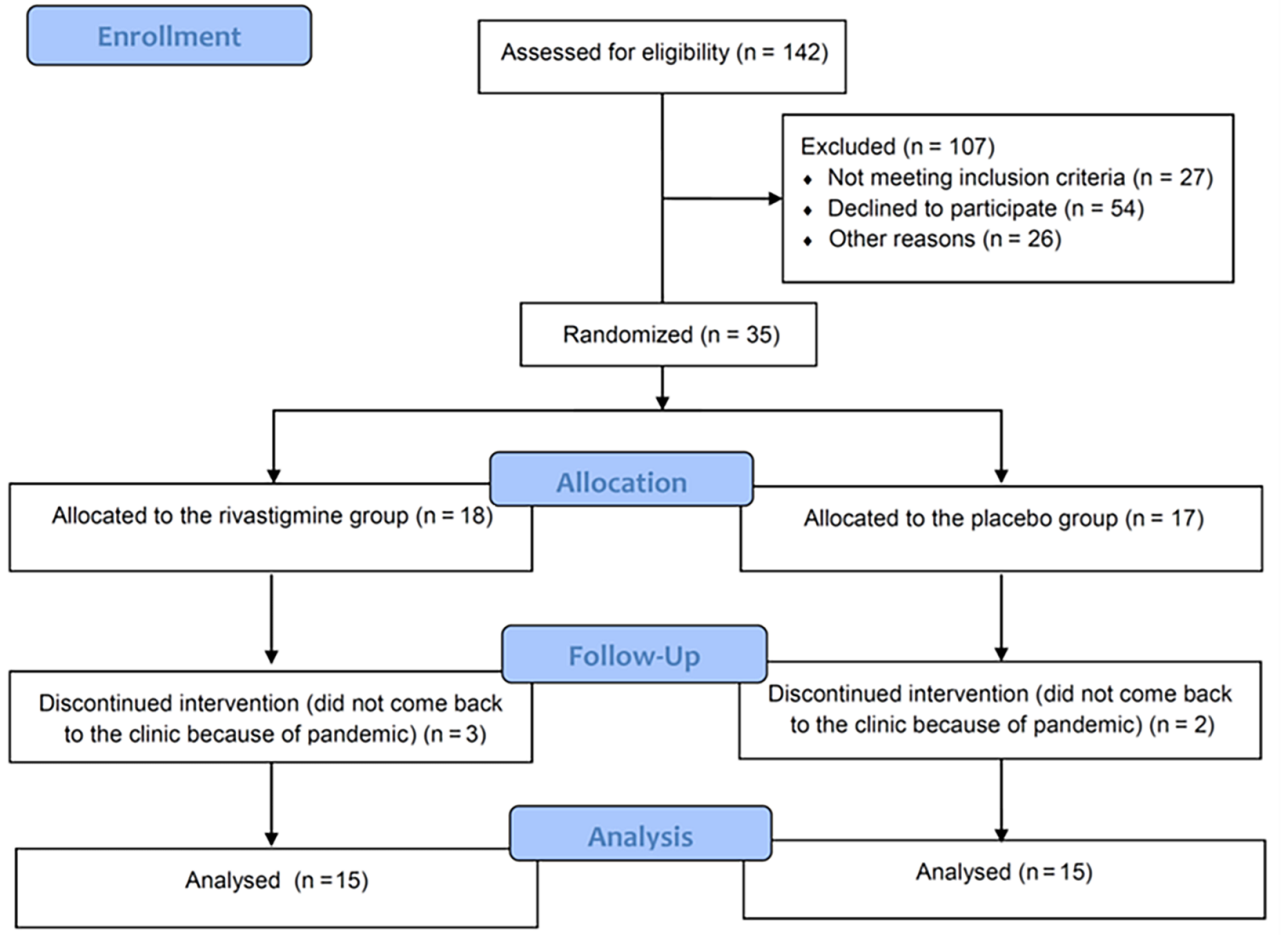
CONSORT diagram of the patients through the study

Among the 142 patients screened, randomization was carried out on 35 patients. Eighteen were assigned to the rivastigmine group, while 17 were assigned to the control group (Figure 1). The study population baseline characteristics are described in Table 2, which shows that the mean age of the patients included in the study in the placebo and rivastigmine groups were 59.33 ± 10.34 years and 58.93 ± 10.88 years, respectively. The age range was 42 to 81 years, and 63% of patients were female. No significant differences were shown between the rivastigmine and the control groups regarding baseline comorbidities such as depression, hypertension, and diabetes mellitus or baseline levels of thyroid stimulating hormone, vitamin B12, or MMSE. Baseline characteristics and lab tests are shown in Table 2. 

**Table 2.** Baseline Characteristics and Laboratory Findings of the Study Population

Baseline Data	Rivastigmine (n = 15)	Placebo (n = 15)	P-Value
**Age, mean ± SD**	58.93 ± 10.88	59.33 ± 10.34	0.91
**Sex (M/F)**	6/9	5/10	1
**MMSE (IQR)**	26 (25 - 26)	26 (25 - 26)	0.23
**Vitamin B12 (pg/mL)**	291 (232 - 449)	321 (302 - 471)	0.62
**TSH (mU/L), mean ± SD**	2.22 ± 0.91	2.26 ± 0.86	0.89

Abbreviations: F, female; IQR, interquartile range; M, male; MMSE, mini-mental state examination; mU/L, milliunits per lite; pg/mL, pictograms per milliliters; SD, standard deviation; TSH, thyroid stimulating hormone.

Rivastigmine significantly improved DM as measured by the tests, as mentioned earlier, compared to placebo. Among the CGT tests, the delay aversion section showed a significant difference in the rivastigmine group compared to the placebo group (P-value = 0.002), meaning patients in the rivastigmine group could make quicker decisions. In the deliberation time section, patients in the rivastigmine group performed better. They could decide based on the available evidence in a shorter period of time, and this difference was statistically significant compared to placebo (P-value = 0.001). In the overall proportion bet section, patients who used the placebo showed a significantly lower mean number that indicated more self-control (P-value = 0.001). In the quality of the DM section, the patients who received rivastigmine had a significantly more logical choice (P-value = 0.001). No significant difference was detected between rivastigmine and placebo regarding the risk-adjustment section (P = 0.12).

As can be seen in the IST test, rivastigmine significantly improved sampling error, and patients in the active arm had a more rational choice (P-value = 0.001). In comparison with placebo, the effects of rivastigmine on discrimination errors were not statistically significant, but in the total correct section, the patients who took rivastigmine performed better (P-value = 0.02).

Regarding SST tests, patients in the rivastigmine group showed significantly lower risk-taking behaviors, which means more self-control (P-value = 0.03). In the direction errors on stop-and-go trials, patients who received the drug performed better with a lower number in the results (P-value = 0.01). In the tests for the proportion of successful stops, which indicates successful stops to total stops, it was found that the patients in the drug group had a significantly better performance (P-value = 0.006). The two groups had no significant difference in the median correct RT on GO trials (P-value = 0.82). There was no significant difference between the two groups in SSRT (last half), showing the stop signal's reaction time (P-value = 0.45). In the SSD (last half) part, the drug group performed better and showed a significant difference (P-value = 0.03) (Table 3). 

**Table 3.** Efficacy Outcomes of the Study ^a^

Test	Rivastigmine (n = 15)	Placebo (n = 15)	P-Value
**CGT**			
Delay aversion	0.38 ± 0.28	0.31 ± 0.51	0.002
Deliberation time	2531.21 ± 1895.06	5523.70 ± 2298.42	0.001
Overall proportion bet	0.58 ± 0.14	0.52 ± 0.15	0.001
Quality of decision making	0.87 ± 0.28	0.68 ± 0.16	0.001
Risk adjustment	0.23 ± 0.47	- 0.041 ± 1.29	0.12
**IST **			
Discrimination errors	0.75 ± 1.54	3.13 ± 1.80	0.19
Sampling errors	2.18 ± 2.08	4.67 ± 1.17	0.001
Total correct	5.62 ± 1.26	4.50 ± 1.22	0.02
**SST **			
Direction errors on stop-and-go trials	0.17 ± 0.38	27.60 ± 30.30	0.01
The proportion of successful stops	0.71 ± 0.19	0.50 ± 0.10	0.006
Median correct RT on GO trials	1106.25 ± 267.41	856.25 ± 130.39	0.82
SSRT (last half)	610.76 ± 262.26	582.26 ± 177.74	0.45
SSD (50%) (last half)	353.41 ± 152.91	273.98 ± 174.94	0.03
Risk-taking	0.54 ± 0.23	0.57 ± 0.18	0.03

Abbreviations: CGT, Cambridge Gambling Task; IST, information sampling task; SD, standard deviation; RT, reaction time; SSD, stop signal delay; SSRT, stop signal reaction time; SST, stop signal test.

^a^ Values are expressed as mean ± SD.

Rivastigmine was tolerated well in this study, and most reported adverse events were gastrointestinal complaints, including gastrointestinal reflux disease (GERD), diarrhea, nausea, vomiting, and anorexia. The placebo group (N = 4) and the rivastigmine group (N = 6) had no significant difference in total gastrointestinal adverse effects (P-value=0.5). Headache and agitation were among the other reported adverse effects of rivastigmine. In total, four out of 15 patients experienced adverse effects in both groups, and the most common reaction was GERD (Table 4). 

**Table 4.** Reported Adverse Drug Reactions ^a^

Symptom	Rivastigmine	Placebo	P-Value
**GERD**	3 (23)	3 (23)	0.63
**Diarrhea**	1 (7)	1 (7)	1
**Nausea**	1 (7)	0 (0)	0.32
**Vomiting**	1 (7)	0 (0)	0.32
**Headache**	1 (7)	0 (0)	0.32
**Agitation**	1 (7)	0 (0)	0.32
**Anorexia**	1 (7)	0 (0)	0.32

Abbreviation: GERD, gastroesophageal reflux disease.

^a^ Values are expressed as No. (%).

Eight cases of adverse effects occurred in the first two weeks, five in the second two weeks, and one in the second month of treatment. There were no adverse effect reports within the third month. Five of the nine reported complications in the rivastigmine group were related to one patient.

One person in the placebo group and two in the rivastigmine group needed medications such as non-steroid anti-inflammatory drugs and proton pump inhibitors to alleviate adverse effects. For two patients in the rivastigmine group, the dose did not increase due to GI disturbances 2 weeks after starting the drug, and the dose escalation was at weeks 3 and 4, respectively.

## 5. Discussion

The current study investigated the effects of rivastigmine administration on DM in patients with MCI. To the best of our knowledge, this is the first RCT that evaluated the effects of this intervention in patients with MCI. Rivastigmine significantly improved the primary outcomes of DM based on CANTAB tests compared to placebo. Analysis of secondary endpoints showed that rivastigmine was tolerated well in this population without any severe adverse events at the dose of 3 mg twice daily.

The progression of dementia in patients aged 65 and older with MCI suspected to be of neurodegenerative origin is estimated at 10% per year and 15% over two years (16). The overall incidence of dementia in the general population at the same age is estimated to be 1 - 3% per year. Gender and level of education have not been consistently shown to predict dementia progression (17). Various studies have been conducted on the effect of drugs on improving cognitive function or delaying the progression of MCI to AD. Methylphenidate, caffeine, nicotine, modafinil, atomoxetine, and, most notably, AChEIs and memantine have shown promising effects on improving symptoms in this population (18). Currently, anti-amyloid monoclonal antibodies are the only medications approved to prevent the progression of MCI or mild AD (19). Since there is still uncertainty regarding the clinical benefits and some major concerns regarding safety and cost issues, the use of this agent is only limited to certain patients.

AChEIs, including donepezil, rivastigmine, and galantamine, have demonstrated beneficial effects in improving episodic memory and attention and had the most significant effect on the frontal and parietal lobes of the brain (20). More detailed assessments using sensitive computerized cognitive tests showed extensive attention, working, and episodic memory improvements. However, in general, the effects of cognitive enhancers such as methylphenidate, modafinil, and AChEIs in healthy subjects appear negligible based on recent systematic reviews (21). All AChEIs often cause gastrointestinal discomfort, possibly leading to drug discontinuation in many patients. These effects could offset any positive aspect of the drug's overall performance. According to the literature review, all other studies showed that in healthy geriatric subjects, rivastigmine could improve learning in motor tasks and connecting symbols and figures, but it can impair verbal and visual episodic memory (22). In the EXACT study conducted by Gauthier et al. in 2005 on 2119 patients with mild to moderate Alzheimer's disease, it was found that taking rivastigmine for six months improved attention, apathy, stress, and agitation, which was linked to an increase of 1.1 points on the MMSE (23). In a 2006 study by Feldman et al., 1018 patients with cognitive impairment randomly received rivastigmine (N = 508) or a placebo (N = 510) and underwent a 48-month follow-up. At the end of the study, the progression of MCI to Alzheimer's in the rivastigmine group was 17.3%. In contrast, it was 21.4% in the placebo group, which did not differ significantly from each other (24).

DM is a frequent and continuous cognitive process and a part of human behavior. It is widely accepted that a frontal brain lobe disturbance can impair decision-making ability. DM is highly related to everyday functioning and autonomy and relies on several cognitive skills, such as semantic and episodic memory and executive functioning. DM impairment may predict MCI and conversion to dementia (8).

A 2013 study by Zois et al. on 80 diabetes mellitus patients with a risk disorder demonstrated people with the risk disorder were more likely to make irrational and risky decisions than healthy volunteers (25). In a study conducted in 2019 on 36 patients with cognitive impairment, 29 patients with AD, and 34 healthy individuals to evaluate DM in conditions of risk and ambiguity with a computer test, it was found that DM in conditions of ambiguity and risk was reduced in both AD and MCI patients. However, DM under risky conditions was reduced only in patients with AD (10).

Some non-pharmacological methods have been used to improve cognitive ability in patients with MCI. According to the reported results, computer-based training greatly affects working memory. It can improve general cognitive results, global cognitive ability, attention, psychosocial performance, verbal memory, and verbal and non-verbal learning (26). Other non-pharmacologic interventions targeting DM have shown promising outcomes in patients with MCI or AD. These include explicit advice, feedback, cognitive training, pleasant rewards, talking mats, and support by caregivers (27). Compared with non-pharmacologic measures, data regarding medication’s effects on DM in patients with cognitive impairment is lacking. Based on our study's results, it seems that patients who take rivastigmine have a better reaction speed and can make better logic-based decisions in less or at least the same amount of time. In some situations, they also have increased risk-taking behavior.

Although, based on our knowledge, this was the first study to evaluate the possible effects of rivastigmine in the MCI population, there were also some limitations regarding its methodology and performance. Because of the limitations regarding the COVID-19 pandemic situation, patient enrollment was conducted at a lower-than-expected rate, and we could not achieve our first target sample size (n = 40). Additionally, nearly 14% of our study population were lost to follow-up and did not return for outcome assessment. Based on our previous experience in the Iranian population, we used the target dose of 3 mg twice daily and assumed it to be tolerated well, and the question of whether higher doses of rivastigmine can have more promising effects or serious adverse events remained unanswered. Altogether, it seems that future studies with a larger sample size and longer follow-up durations in different populations from multiple centers evaluating the effects of AChEIs on DM may answer many questions more precisely. Moreover, since there is more robust data regarding the effectiveness of non-pharmacologic therapies on decision-masking, evaluating the role of combination therapies with pharmacological and non-pharmacological treatments could be favorable for patients with MCI.

### 5.1. Conclusions

Based on the current study, among patients with MCI, those who took rivastigmine showed better DM abilities than those who took a placebo. Rivastigmine resulted in better reaction speed (P-value = 0.006), SSD (P-value = 0.03), and more logic-based decision-making (P-value = 0.03). In some situations, patients in the rivastigmine group performed better at risk-taking behaviors.

## Clinical Trial Registration Code:

This study was registered on the Clinical Trial Database on May 10, 2021 (IRCT20201104049257N1).
